# Generative artificial intelligence use in evidence synthesis: A systematic review

**DOI:** 10.1017/rsm.2025.16

**Published:** 2025-04-24

**Authors:** Justin Clark, Belinda Barton, Loai Albarqouni, Oyungerel Byambasuren, Tanisha Jowsey, Justin Keogh, Tian Liang, Christian Moro, Hayley O’Neill, Mark Jones

**Affiliations:** 1 Institute for Evidence-Based Healthcare, Bond University, Gold Coast, QLD, Australia; 2 Bond Business School, Bond University, Gold Coast, QLD, Australia; 3 Faculty of Health Sciences and Medicine, Bond University, Gold Coast, QLD, Australia

**Keywords:** automation, evidence synthesis, generative artificial intelligence (GenAI), large language models (LLMs), systematic reviews

## Abstract

**Introduction:**

With the increasing accessibility of tools such as ChatGPT, Copilot, DeepSeek, Dall-E, and Gemini, generative artificial intelligence (GenAI) has been poised as a potential, research timesaving tool, especially for synthesising evidence. Our objective was to determine whether GenAI can assist with evidence synthesis by assessing its performance using its accuracy, error rates, and time savings compared to the traditional expert-driven approach.

**Methods:**

To systematically review the evidence, we searched five databases on 17 January 2025, synthesised outcomes reporting on the accuracy, error rates, or time taken, and appraised the risk-of-bias using a modified version of QUADAS-2.

**Results:**

We identified 3,071 unique records, 19 of which were included in our review. Most studies had a high or unclear risk-of-bias in Domain 1A: review selection, Domain 2A: GenAI conduct, and Domain 1B: applicability of results. When used for (1) searching GenAI missed 68% to 96% (median = 91%) of studies, (2) screening made incorrect inclusion decisions ranging from 0% to 29% (median = 10%); and incorrect exclusion decisions ranging from 1% to 83% (median = 28%), (3) incorrect data extractions ranging from 4% to 31% (median = 14%), (4) incorrect risk-of-bias assessments ranging from 10% to 56% (median = 27%).

**Conclusion:**

Our review shows that the current evidence does not support GenAI use in evidence synthesis without human involvement or oversight. However, for most tasks other than searching, GenAI may have a role in assisting humans with evidence synthesis.

## Highlights

### What is already known

There is great interest in using Generative Artificial Intelligence (GenAI) as a research time-saving tool. Although GenAI is being used in research, little is known about how frequently it is used or which aspects of research it supports.

### What is new

Despite GenAI being promoted as a research timesaver there is little evidence that supports this and most of the evidence that does exist is not as robust as it should be.

This review indicates that the available evidence suggests GenAI is not currently suitable to for use in evidence synthesis without caution and human oversight as it makes a substantial number of mistakes during evidence synthesis tasks.

### Potential impact for *RSM* readers

This review assesses and quantifies the errors GenAI makes during evidence synthesis, providing much-needed evidence to help researchers decide whether to use GenAI in their own projects.

This review also clearly highlights the need for robust, high-quality studies to be conducted on the safety of using GenAI in research.

## 1 Introduction

Generative artificial intelligence (GenAI) includes a wide spectrum of artificial intelligence (AI) dedicated to creating or generating content or data that frequently resembles human-generated content. While evidence synthesis has traditionally been an expert-driven pursuit, the use of AI^1^—or GenAI—could speed up processes, enhance critical appraisal, facilitate evaluation, and assist experts with writing. There is a burgeoning array of products entering the market to support evidence synthesis and appraisal, such as ASReview,^2^ Covidence,^3^ SciSpace Literature Review,^4^ and Elicit.^5^ A recent study reported significant time savings associated with using artificial intelligence during the screening stages.^2^ A recent review of AI in evidence synthesis identified 12 reviews that used nine tools to implement 15 different artificial intelligence methods—eleven methods for screening, two for data extraction, and two for risk-of-bias assessment. This shows artificial intelligence is being used with varying success. However, further evaluation is required to determine the overall contributions in terms of efficiency and quality.^1^

Considerable scepticism remains about the accuracy, bias, ethics, and effectiveness of using GenAI for evidence synthesis and appraisal. Some researchers warn that current GenAI products for evidence synthesis often lack usability and user-friendliness, hindering their acceptance within the wider research community.^6^ They note that amidst the AI revolution affecting numerous fields, human critical thinking and creativity remain indispensable and continue to be central to the responsibilities of researchers.^6^ Meanwhile, in response to the increased capability and popularity of GenAI demonstrated with the launch of Chat-GPT, there has been a rapid influx of published literature, particularly since December 2022: demonstrating an ever-increasing need to identify and synthesise evidence.

Although GenAI for evidence synthesis is an emerging field, its rapid expansion nature means that by identifying good practices early on, researchers can confidently incorporate it into various evidence synthesis processes. In this review, we aimed to examine currently available evidence of the impact of GenAI in evidence synthesis. We included studies that compared traditional expert-driven approaches to evidence synthesis with GenAI approaches. Our research question was: what are the accuracy, error rates, or time savings associated with using GenAI to conduct evidence synthesis tasks?

## 2 Methods

### 2.1 Protocol and registration

This review followed the 2020 Reporting Items for Systematic Reviews and Meta-Analysis (PRISMA) guidelines.^7^ The study protocol was registered with Open Science Framework on the 3rd of June 2024 (https://osf.io/uytfd/?view_only=1d57a8bb47c74155ab1e853902d05ac6). A PRISMA checklist is available in Appendix 1 of the Supplementary Material.

### 2.2 Eligibility criteria

We included published, peer-reviewed, (e.g., journal articles and conference papers were included while preprints and conference abstracts were excluded), comparative studies where tasks required for evidence synthesis (e.g., full systematic reviews or individual components like screening or data extraction) were fully conducted (excluding planning tasks, e.g., tasks 3 to 19 from the two-week systematic review methodology^8^ were included) where GenAI performance was compared to human performance (e.g., humans conducting an evidence synthesis task, (e.g., the number of correct include/exclude decisions made during title/abstract screening. The outcomes varied across tasks but were broadly classified into accuracy, error rates, or time. We included studies conducted in any research discipline (e.g., medicine and business).^8^

### 2.3 Search strategy

The database search was initially designed in PubMed by an experienced information specialist. It was translated to be run in the other databases: Embase, Web of Science, Scopus, and Business Source Ultimate using the Polyglot Search Translator.^9^ Full search strings for all databases are available in Appendix 2 of the Supplementary Material. The searches were run from inception until 17 January 2025. No language restrictions were applied to the search. A backward and forward citation search was conducted on 18 June 2024 using the SpiderCite tool.^8^

### 2.4 Study selection and screening

Study authors (T.J., B.B., T.L., H.N., M.J., J.K., C.M., J.C., H.N., L.A., and M.J.) worked in pairs to independently screen each record against the eligibility criteria, this was for both title/abstract and full-text screening.

### 2.5 Data extraction

A standardised form (initially piloted on three included studies) was used for data extraction of characteristics of studies, outcomes, and risk of bias. Study authors (T.J., B.B., T.L., H.N., M.J., J.K., C.M., J.C., and M.J.) worked in pairs to independently extract the following data from included studies:

- types: comparative study.
- methods: study authors, year, country, study design, and setting.
- participants: type of evidence synthesis, number of reviews used/searches done/records screened/data extracted/risk-of-bias domains assessed.
- interventions and comparators: evidence synthesis task, GenAI or LLM model/type/program, who provided the intervention, type of comparator.
- outcomes: accuracy, error rates, and time.

### 2.6 Assessment of the risk of bias

The risk-of-bias was assessed using a modified version of the QUADAS-2 tool.^10^ Study authors (O.B., M.J., J.C., J.K., T.L., T.J., and H.O.) worked in pairs to independently assess the risk-of-bias for each study.

QUADAS-2 is a tool for assessing the quality of diagnostic accuracy studies and was selected due to the similarities between evaluating diagnostic tests and evaluating the accuracy of conducting systematic review tasks.

*Modified QUADAS-2 for assessing the risk-of-bias in GenAI comparative studies*

*Domain 1: Review selection*

Domain 1A Risk of bias: Could the selection of reviews have introduced bias?

Domain 1B Concerns regarding applicability: Is there a concern that the study findings in the evaluations may not be applicable to all types of review? (e.g., only searching for a single study type or sample size too small).

*Domain 2: GenAI conduct*

Domain 2A Risk of bias: Could the conduct or interpretation of the GenAI test have introduced bias?

Domain 2B Concerns regarding replicability: Is there concern that the GenAI/LLM tool cannot be used effectively by a standard review team?

*Domain 3: Human conduct*

Domain 3A Risk of bias: Could the conduct or interpretation of human performance have introduced bias?

Domain 3B Concerns regarding replicability: Is there concern that the review task/s performed by humans cannot be replicated by a standard SR team?

*Domain 4: Differences.*

Domain 4 Risk of bias: Could any differences between the GenAI and human conduct have introduced bias?

A comparison between the original QUADAS-2 and our modified version can be found in Appendix 3 of the Supplementary Material.

### 2.7 Measurement of effect

The measures used to evaluate performance varied across the different tasks.

Designing searches was measured using:

- Recall = the percentage of relevant records found, divided by the total number of relevant records available, for example, if there are 10 relevant reports that could be found and the search finds 8 of them, it has a recall of 80%.
- Precision = the number of relevant records found divided by the total number of records retrieved, for example, if a search retrieves 1,000 records and 8 of them are relevant, the precision is 0.008.
- Number needed to read (NNR) = one divided by precision, for example, if a search retrieves 1,000 records and 8 of them relevant, the precision is 0.008, resulting in an NNR of 125, in other words, you need to screen 125 records to find 1 relevant record.
- Errors = the percentage of relevant records not found, for example, 100%—recall.

Screening (title/abstract and full text) studies were measured using:

- Relevant studies included = the percentage of relevant records that should have been included in the review and were included.
- Relevant studies excluded = the percentage of relevant records that should have been included in the review but were incorrectly excluded.
- Irrelevant studies excluded = the percentage of irrelevant records that should have been excluded from the review and were correctly excluded.
- Irrelevant studies included = the percentage of irrelevant records that should have been excluded from the review and were incorrectly included.
- Errors = incorrect excludes summed with incorrect includes.

Data extraction was measured using:

- Correct extraction = the number of correctly extracted data or correctly identifying the data was missing from the manuscript.
- Incorrect extraction = the number of incorrectly extracted data or not identifying that the data was missing from the manuscript.
- Errors = the percentage of incorrectly extracted data or not identifying that the data was missing from the manuscript.

The risk-of-bias was measured using:

- Correct assessment = the number of correctly assessed risk-of-bias domains or identifying this information was missing from the manuscript.
- Incorrect assessment = the number of incorrectly assessed risk-of-bias domains or not identifying this information was missing from the manuscript.
- Errors = the percentage of incorrectly assessed risk-of-bias domains or not identifying this information was missing from the manuscript.

### 2.8 Unit of analysis

The unit of analysis was records or reports for searching and screening, data elements within a manuscript for data extraction, and risk-of-bias domains within a manuscript for risk of bias.

### 2.9 Dealing with missing data

We did not contact investigators or study sponsors to provide missing data.

### 2.10 Assessment of heterogeneity

Heterogeneity was expected to be high therefore we stratified our synthesis by systematic review task (searching, abstract screening, full-text screening, data extraction, and risk of bias). As the data was not amenable to being meta-analysed, we synthesised the data narratively.

### 2.11 Assessment of publication biases

We did not assess publication bias/small studies effect because fewer than 10 studies were included for each systematic review task.

## 3 Results

### 3.1 Results of the search

**Figure 1:**
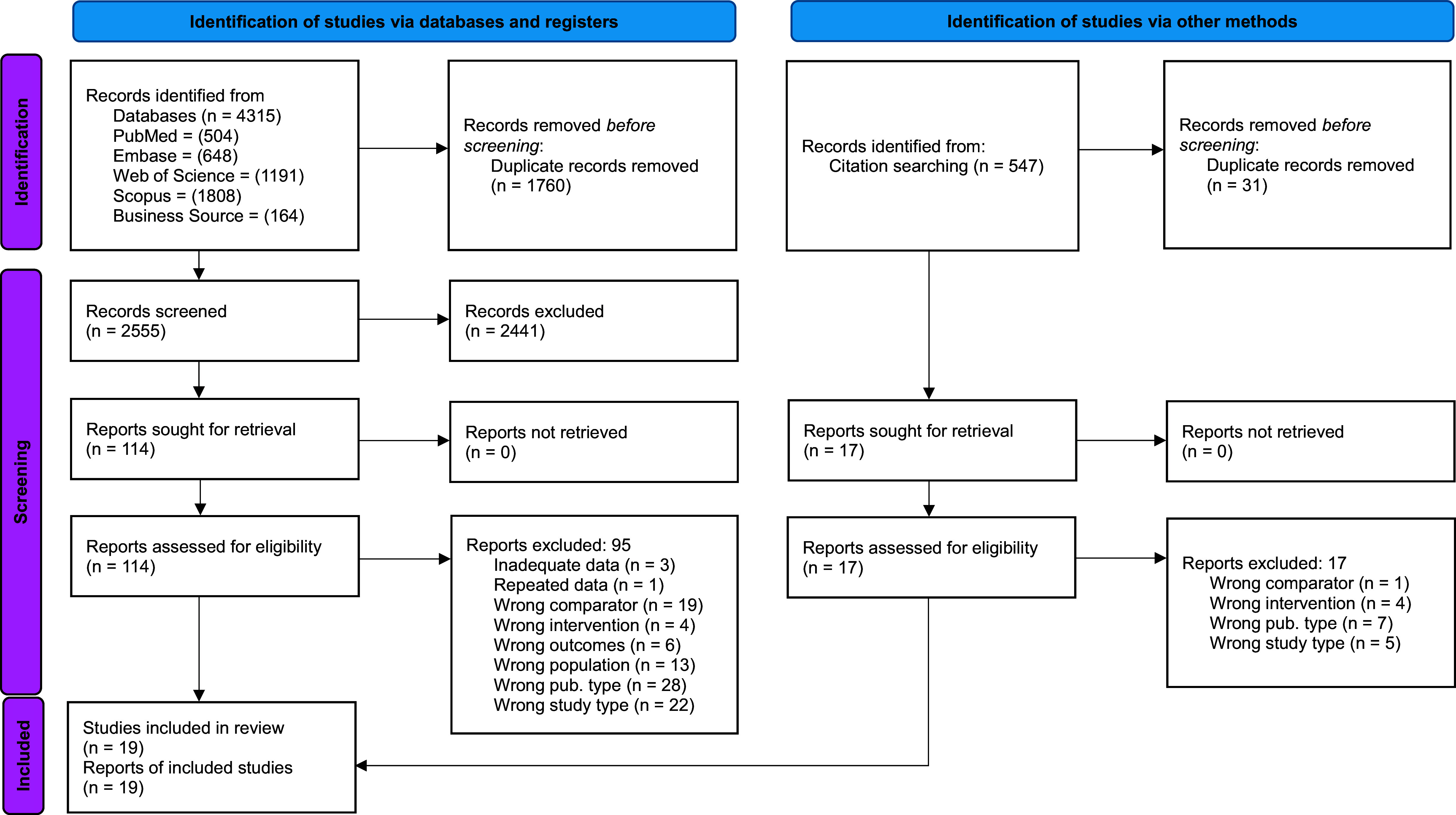
PRISMA 2020 flow diagram of study inclusion.

We retrieved a total of 4,862 records from the database and citation searches. After duplicate records were removed, 3,071 unique records remained which were screened for eligibility. During title/abstract screening, 2,971 were excluded leaving 131 for full text screening. During full-text screening, 112 studies were excluded leaving 19 studies for inclusion in this review (Figure 1).^7^ A full list of excluded studies is available in Appendix 4 of the Supplementary Material.

## 4 Characteristics of included studies

All studies were published in the last two years, from a mix of countries from North America, Europe, the Middle East, and Asia. Settings were mostly health apart from two studies in information technology. The GenAI tools assessed included chat generative pre-trained transformers (ChatGPT, GPT, Claude, Bing AI, and Perplexity.AI). Systematic review tasks assessed were searching, screening, data extraction, and risk-of-bias assessment. Most studies compared the GenAI tools to already published systematic reviews conducted by humans (Table 1).

**Table 1:** Characteristics of included studies

Study ID	Country	Setting	Model/s	Comparator	Task/s
Gwon et al.^11^	South Korea	Health	Bing AI and ChatGPT	Expert reviewer	Searching
Sanii et al.^12^	USA	Health	ChatGPT and Perplexity.AI	Consensus between two reviewers	Searching
Wang et al.^13^	Australia	Health	ChatGPT	Expert searcher	Searching
Felizardo et al.^14^	Brazil	Computer Science	ChatGPT–4.0	Consensus between two reviewers	Title/abstract screening
Guo et al.^15^	Canada	Health	ChatGPT	Consensus between two reviewers	Title/abstract screening
Issaiy et al.^16^	Iran	Health	ChatGPT	Consensus between three expert reviewers	Title/abstract screening
Matsui et al.^17^	Japan	Health	GPT–3.5 and GPT–4	Consensus between two reviewers	Title/abstract screening
Schopow et al.^18^	Germany	Health	ChatGPT–3.5 legacy	Consensus between two reviewers	Title/abstract screening
Tran et al.^19^	France	Health	ChatGPT	Consensus between two reviewers	Title/abstract screening
Khraisha et al.^20^	Ireland	Health	GPT–4	Consensus between two reviewers	Title/abstract screening, Full-text screening and data extraction
Oami et al.^21^	Japan	Health	GPT–4 Turbo	Consensus between two reviewers	Screening (title/abstract and full text combined)
Strachan et al.^22^	Scotland	Health	GPT–3	Consensus between two reviewers	Screening (title/abstract and full text combined)
Felizardo et al.^23^	Brazil	Computer Science	ChatGPT–4.0	Consensus between two reviewers	Data extraction
Gartlehner et al.^24^	USA	Health	Claude 2	Single reviewer checked by a second reviewer	Data extraction
Jensen et al.^25^	Denmark	Health	ChatGPT–4o	Consensus between three reviewers	Data extraction
Konet et al.^26^	USA	Health	Claude 2 and ChatGPT–4	Compared to the published review	Data extraction
Hasan et al.^27^	USA	Health	GPT–4	Consensus between two reviewers	Risk-of-bias assessment
Lai et al.^28^	China	Health	ChatGPT and Claude	Consensus between three expert reviewers	Risk-of-bias assessment
Tarakji et al.^29^	USA	Health	GPT–4	Single reviewer checked by a second reviewer	Risk-of-bias assessment

### 4.1 Risk of bias

**Figure 2:**
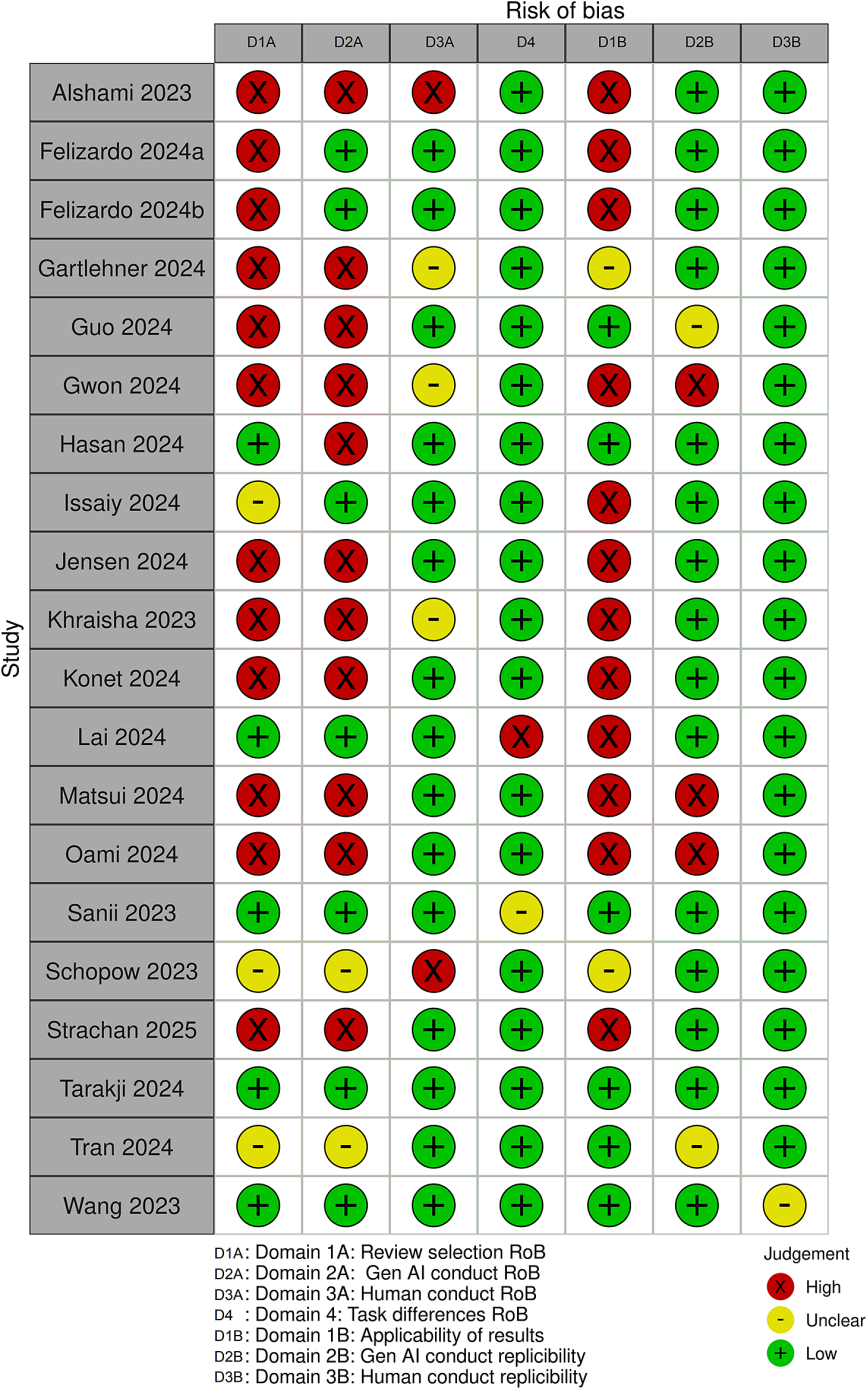
Individual study risk of bias.

**Figure 3:**
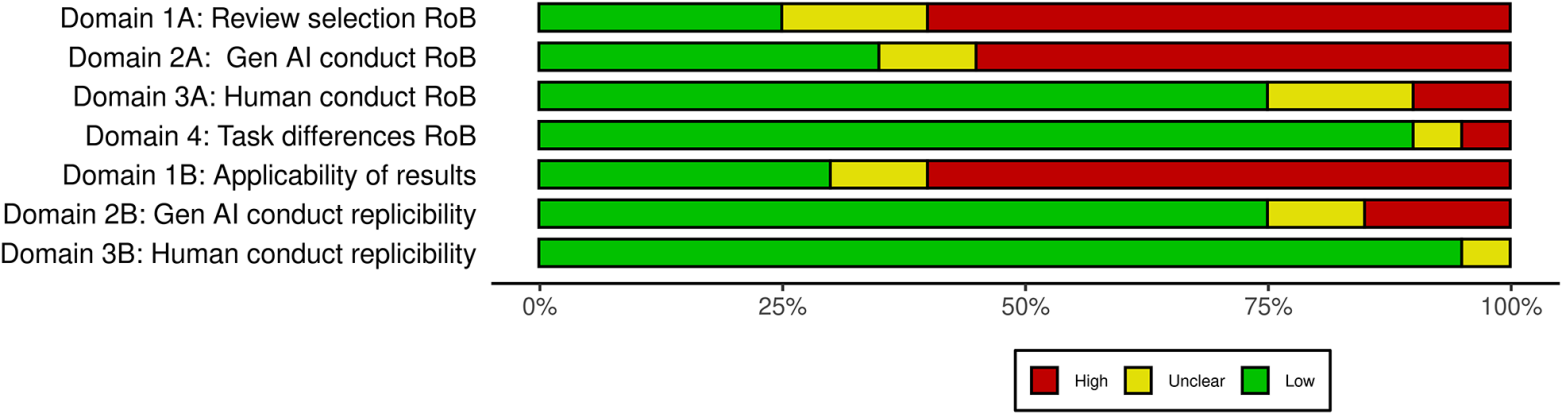
Domain summary of the risk of bias.

Risk-of-Bias was assessed using a modified version of the QUADAS-2 tool^10^ and presented in the manuscript using the Risk-of-bias VISualization (robvis) tool^30^ (Figure 2). Most studies had a high or unclear risk-of-bias in Domain 1A: review selection, domain as most studies either included a single review or used a convenience sample, Domain 2A: GenAI conduct mostly due to the fact study authors already knew the results of the tasks they were asking GenAI to do, or they modified prompts during an evaluation phase to maximise GenAI performance and Domain 1B: applicability of results, primarily due to the small sample size or the restricted topics in the sample (Figure 3).

### 4.2 GenAI for designing searches

Three studies evaluated GenAI for conducting literature search tasks in evidence synthesis.^11^ ^–^ ^13^ All three assessed the recall (percentage of relevant studies found) which ranged from 4% to 32%, with an average of 13%. This means GenAI tools missed between 68% and 96% of the relevant studies available that were found by humans. Precision reported as the NNR was evaluated in two of the studies,^11^ ^,^ ^13^ revealing that the NNR from 9 to 1,287, with an average of 14. In comparison, the corresponding numbers for humans showed a considerably smaller range, between 9 and 35. One study^12^ reported the time needed to design searches using GenAI tools which ranged from five to 57 minutes, while humans took 644 minutes (Table 2).

**Table 2:** Outcomes stratified by evidence synthesis task

Searching study ID	Model/method used		*N* (s) (number of searches)	Errors % (relevant studies missed)*	Precision (number needed to read)	Time (minutes)		
Gwon et al.^11^	Human (comparator)		1	0%	9			
	ChatGPT		1	96%	1,287			
	BingAI		1	82%	24			
Sanii et al.^12^	Human (comparator)		5	0%		644		
	ChatGPT		5	95%		5		
	Perplexity.AI		5	82%		57		
Wang et al.^13^	Human (comparator)		112	0%	35			
	ChatGPT Prompt 1 (q1)		112	91%	19			
	ChatGPT Prompt 2 (q2)		112	91%	9			
	ChatGPT Prompt 3 (q3)		112	92%	13			
	ChatGPT Prompt 4 (q4)		112	68%	19			
	ChatGPT Prompt 5 (q5)		112	79%	17			

Title/abstract screening study ID	Model/method used	*N* (r) (number of reviews)	*N* (a) (number of records screened)	Errors % (records incorrectly included or excluded)*	Relevant studies included	Relevant studies excluded	Irrelevant studies excluded	Irrelevant studies included
Felizardo et al.^14^	Human (comparator)	2	582	0%	212	0	370	0
	ChatGPT	2	582	19%	76% (161)	24% (51)	83% (326)	17% (44)
Guo et al.^15^	Human (comparator)	6	24,844	0%	538	0	24,305	0
	Chat GPT	6	24,844	12%	81% (411)	19% (127)	90% (22,129)	10% (2,176)
Issaiy et al.^16^	Expert humans (comparator)	3	1,198	0%	148	0	1,050	0
	Non-expert humans	3	1,198	6%	62% (92)	38% (56)	98% (1,031)	2% (19)
	ChatGPT (threshold ≥3)	3	1,198	31%	95% (140)	5% (8)	65% (684)	35% (366)

Khraisha et al.^20^	Human (comparator)	1	300	0%				
	GPT4 (English and reviewed)	1	100	33%				
	GPT4 (English and grey)	1	100	34%				
	GPT4 (Other languages)	1	100	22%				
Matsui et al.^17^	Human (comparator)	2	4,527	0%	126	0	4,401	0
	GPT3.5	2	4,527	57%	93% (119)	7% (7)	41% (1,310)	59% (3,091)
	GPT4	2	4,527	8%	84% 108)	16% (18)	85% (3,952)	15% (449)
Schopow et al.^18^	Human (comparator)	1	155	0%	41	0	114	0
	ChatGPT3.5 legacy (Abstract)	1	155	43%	100% (41)	0% (0)	41% (47)	59% (67)

Tran et al.^19^	Human (comparator)	5	22,665	0%	1,926	0	20,739	0
	ChatGPT (Balanced)	5	22,665	43%	87% (1,756)	13% (170)	52% (10,460)	48% (10,279)
	ChatGPT (Sensitive)	5	22,665	71%	98% (1,911)	2% (15)	17% (3,409)	83% (17,330)

Full-text screening study ID	Model/method used	*N* (r) (number of reviews)	*N* (a) (number of records screened)	Errors % (records incorrectly included or excluded)*	Relevant studies included	Relevant studies excluded	Irrelevant studies excluded	Irrelevant studies included
Khraisha et al.^20^	Human (comparator)	1	150	0%				
	GPT4 (English and reviewed)	1	50	46%				
	GPT4 (English and grey)	1	50	22%				
	GPT4 (Other languages)	1	50	4%				
				Errors %				
Title/abstract				(reports				
and full text			*N* (a) (number	incorrectly	Relevant	Relevant	Irrelevant	Irrelevant
screening	Model/method	*N* (r) (number	of reports	included or	studies	studies	studies	studies
study ID	used	of reviews)	screened)	excluded)*	included	excluded	excluded	included
Oami et al.^21^	Human (comparator)	5	16,669	0%	41	0	16,628	0
	GPT–4 Turbo	5	16,669	1%	71% (33)	29% (8)	99% (16,495)	1% (133)
Strachan^22^	Human (comparator)	3	1,977	0%	32		1945	
	GPT 3	3	1,977	21%	99% (31)	1% (1)	79% (1,570)	21% (375)

Data extraction study ID	Model/method used	*N* (s) (number of studies)	*N* (d) (number of data elements extracted)	Errors % (incorrectly or not extracted data)*	Correct extraction	Incorrect extraction		
Felizardo et al.^23^	Human (comparator)	25	370	0%	370	0		
	ChatGPT	25	370	12%	325	45		
Gartlehner et al.^24^	Human (comparator)	10	157	0%	157	0		
	Claude 2	10	157	4%	151	6		
Jensen et al.^25^	Human (comparator)	11	484	0%	484	0		
	ChatGPT4	11	484	8%	447	37		
Khraisha et al.^20^	Human (comparator)	30		0%				
	GPT4 (English and reviewed)	16		18%				
	GPT4 (English and grey)	10		19%				
	GPT4 (Other languages)	4		15%				

Konet et al.^26^	Human (comparator)	10	160	0%	160	0		
	Claude 2	10	160	4%	154	6		
	ChatGPT	10	160	31%	111	49		

Assessing risk-of-bias study ID	Model/method used	*N* (s) (number of studies)	*N* (RoB) (RoB domains assessed)	Errors % (Incorrectly or not assessed domains)*	Correct assessment	Incorrect assessment		
Hasan et al.^27^	Human (comparator)	307	2,149	0%				
	GPT4	307	2,149	56%				
Lai et al.^28^	Human (comparator)	30	300	0%	300	0		
	ChatGPT (LLM 1)	30	300	15%	253	47		
	Claude (LLM 2)	30	300	10%	268	32		

Tarakji et al.^23^	Human (comparator)	797	6,376	0	6,376	0		
	ChatGPT4	797	6,376	40%	3,795	2,581		

* Average errors were calculated by averaging across individual study errors.

### 4.3 GenAI for title/abstract screening

Seven studies assessed the accuracy of GenAI for title/abstract screening.^14^ ^–^ ^20^ The number of articles screened ranged from 100 to 24,844, while the error rate (records incorrectly included or excluded) ranged from 8% to 71% with a median of 34% (Table 2).

### 4.4 GenAI for full-text screening

One study assessed the accuracy of GenAI for full-text screening.^20^ It assessed full-text screening across three scenarios, 1) peer-reviewed records published in English; 2) grey literature; and 3) peer-reviewed records published in languages other than English. The number of reports screened was 50 for each scenario and the number of errors (reports incorrectly included or excluded) ranged from 4% to 46% (Table 2).^20^

### 4.5 GenAI for Title/abstract and full-text screening

Two studies assessed the accuracy of GenAI for the combined process of title/abstract and full-text screening.^21^ ^,^ ^22^ The number of records screened ranged from 1,977 to 16,669 while the number of errors made (records incorrectly included or excluded) ranged from 1% to 21% (Table 2).

### 4.6 GenAI for data extraction

Five studies assessed the accuracy of GenAI for data extraction.^20^ ^,^ ^23^ ^,^ ^24^ The number of elements extracted ranged from 157 to 484, while errors ranged from 4% to 31% (Table 2).

### 4.7 GenAI for risk-of-bias assessment

Three studies assessed the accuracy of using GenAI for risk-of-bias assessment.^27^ ^–^ ^29^ The number of risk-of-bias domains assessed ranged from 300 to 6,376, while error rate ranged from 10% to 56%. One study focused on assessing the risk-of-bias in randomised trials,^28^ while the other two examined it in observational studies.^27^ ^,^ ^29^ GenAI performed substantially better at assessing the risk-of-bias in trials with average errors ranging from 10% to 15% compared to observational studies, with errors ranging from 40% to 56% (Table 2).

## 5 Discussion

A fundamental cornerstone of health sciences and other disciplines is the systemic review and synthesis of existing research to answer new policy, clinical, and other questions. While GenAI may eventually improve productivity in evidence synthesis, our review highlights that the technology is not yet reliable for tasks such as searching and making inclusion/exclusion decisions. GenAI shows promise for some of these tasks but only with continued expert human oversight, which is still the gold standard when synthesising evidence. While scientists may be excited about the potential time savings associated with GenAI for science and scholarship, only one reviewed study reported time savings with searching, but these saving were not accompanied by reliable GenAI outputs. This suggests that we made a wise decision not to use GenAI to assist in conducting this review.

Although GenAI tools may eventually enhance productivity, our systematic review reveals that the evidence supporting them lacks robustness. Our findings show that the current accuracy of outputs from GenAI tools often falls short of the standards achieved by human researchers, sometimes far short. This is especially seen in both the searching and screening tasks where it is not currently good enough to be used. GenAI demonstrated better performance in tasks such as data extraction and assessing risk-of-bias in some domains, but not all. For data extraction, GenAI performed well in what could be considered the “easier” data to extract, such as publication years or countries, or where numbers were involved, for example, participant numbers. For more complex data, such as outcome data or intervention descriptions, GenAI tended to perform less effectively. Similar results were observed in risk-of-bias assessment. Again, GenAI performed well in the “easier” task of assessing the risk-of-bias for randomised trials, but struggled with more complex task of assessing bias in non-randomised studies. Additionally, many studies in our systematic review had a high risk-of-bias across multiple domains, further obscuring the potential benefits GenAI for evidence synthesis.

To the best of our knowledge, there is currently no published and peer-reviewed systematic review on the topic of GenAI performance for synthesising evidence. Therefore, setting our work in the context of current research is challenging. Although no systemic review exist, several published opinions address the topic. Some opinion pieces raise valid concerns about the impact GenAI could have on the research integrity, generally by producing large quantities of low-quality research quickly, and therefore recommend developing and following guidelines on the appropriate use of GenAI in evidence synthesis.^31^ ^–^ ^33^ Other opinion pieces, typically written by scientists using GenAI tools, suggest that it can already be extremely useful. While these opinions may be valid, there is no peer-reviewed evidence to support them.^4^ ^,^ ^5^ Our findings suggest that although there may be opportunities for GenAI to assist, these need to be tempered with a clear understanding of both the strengths of GenAI and its weaknesses. Some of these strengths and weaknesses have been summarised in a recent commentary.^6^ They identified strengths in developing topic descriptions, exploring those topics, and potentially obtaining GenAI summaries. This could help with the planning of a review. Some concerns were dilemmas around authorship and therefore responsibility for the content GenAI creates, and of course the issue of misinformation where GenAI hallucinates and provides incorrect or fake information and references.^6^

### 5.1 Limitations

The primary limitation faced by this review is the speed with which advances are being made in GenAI technology. There is always a delay between the evaluation of new technologies or methodologies and the publication of the results. We have sought to mitigate this limitation by updating our search shortly before the submitting our manuscript for publication. As GenAI is anticipated to continue improving, it is recommended that any additional recent publications are included when considering the findings of this systematic review in subsequent years. Another challenge was the pioneering nature of our review, which required us to adapt an existing risk-of-bias-tool (QUADAS 2), to assess the quality of the relevant studies. Our review is also limited by the surprisingly small number of relevant studies (*n* = 19), the variable quality of the studies, the limited number of studies evaluating each systematic review task (ranging from one to seven), and the inconsistent reporting across studies. Our last limitation is we only included studies that directly compared GenAI to humans. This means any studies conducting a retrospective analysis against existing datasets of review tasks could have been missed and not included in our synthesis. Also, we did not include studies that conducted only a part of a review task, for example, only extracting the PICO of a study. This may lead to actual GenAI performance in certain tasks being understated by our results.

## 6 Conclusions

The findings of this review underscore that, despite the rapid technological advances in GenAI, the evidence shows it is not yet ready to be used in evidence synthesis without human oversight. We recommend that researchers do not use GenAI tools for searching. We recommend caution and human oversight if it is used for screening, data extraction, or risk-of-bias assessment. Given the rapid pace of development in this field, we recommend that the literature be systematically reviewed at regular intervals, possibly annually, to update the findings presented here. We also highly recommend that evaluations of GenAI tools be conducted before they are used for evidence synthesis.

## Supporting information

Clark et al. supplementary material Clark et al. supplementary material

## Data Availability

Additional data are available via the Open Science Framework (OSF) at https://osf.io/rmyz3/?view_only=609e2fec7f3842a6b4c4a4f176515307.

## Supplementary material

To view supplementary material for this article, please visit http://doi.org/10.1017/rsm.2025.16.
