# Supplementary material for: Generative artificial intelligence use in evidence synthesis: A systematic review
Source: Res Synth Methods. 2025 Apr 24;16(4):601–19. doi: 10.1017/rsm.2025.16 (PMC12527500; doi:10.1017/rsm.2025.16)
Supplement: Clark et al. supplementary material [file S175928792500016Xsup001.docx]

# Generative artificial intelligence use in evidence synthesis: a systematic review

Justin Clark^1^*, Belinda Barton^2^, Loai Albarqouni^1^, Oyungerel Byambasuren^1^, Tanisha Jowsey^3^, Justin Keogh^3^, Tian Liang^1^, Christian Moro^3^, Hayley O'Neill^3^, Mark Jones^1^.

* Corresponding author Justin Clark: [jclark@bond.edu.au](mailto:jclark@bond.edu.au); 14 University Drive, Robina, Qld, Australia, 4226, Phone: +61 7 55955545

1 Institute for Evidence-Based Healthcare, Bond University, Gold Coast, Australia

2 Bond Business School, Bond University, Gold Coast, Australia

3 Faculty of Health Sciences and Medicine, Bond University, Gold Coast, Australia

# Appendix (supplementary materials)

| **Section and Topic** | **Item #** | **Checklist item** | **Location where item is reported** |
| --- | --- | --- | --- |
| **TITLE** | | |  |
| Title | 1 | Identify the report as a systematic review. | 1 |
| **ABSTRACT** | | |  |
| Abstract | 2 | See the PRISMA 2020 for Abstracts checklist. | 2 |
| **INTRODUCTION** | | |  |
| Rationale | 3 | Describe the rationale for the review in the context of existing knowledge. | 4 |
| Objectives | 4 | Provide an explicit statement of the objective(s) or question(s) the review addresses. | 4 |
| **METHODS** | | |  |
| Eligibility criteria | 5 | Specify the inclusion and exclusion criteria for the review and how studies were grouped for the syntheses. | 5 |
| Information sources | 6 | Specify all databases, registers, websites, organisations, reference lists and other sources searched or consulted to identify studies. Specify the date when each source was last searched or consulted. | 5 |
| Search strategy | 7 | Present the full search strategies for all databases, registers and websites, including any filters and limits used. | Appendix 2 |
| Selection process | 8 | Specify the methods used to decide whether a study met the inclusion criteria of the review, including how many reviewers screened each record and each report retrieved, whether they worked independently, and if applicable, details of automation tools used in the process. | 5 |
| Data collection process | 9 | Specify the methods used to collect data from reports, including how many reviewers collected data from each report, whether they worked independently, any processes for obtaining or confirming data from study investigators, and if applicable, details of automation tools used in the process. | 6 |
| Data items | 10a | List and define all outcomes for which data were sought. Specify whether all results that were compatible with each outcome domain in each study were sought (e.g. for all measures, time points, analyses), and if not, the methods used to decide which results to collect. | 6 |
|  | 10b | List and define all other variables for which data were sought (e.g. participant and intervention characteristics, funding sources). Describe any assumptions made about any missing or unclear information. | 6 |
| Study risk of bias assessment | 11 | Specify the methods used to assess risk of bias in the included studies, including details of the tool(s) used, how many reviewers assessed each study and whether they worked independently, and if applicable, details of automation tools used in the process. | 6 |
| Effect measures | 12 | Specify for each outcome the effect measure(s) (e.g. risk ratio, mean difference) used in the synthesis or presentation of results. | 6-7 |
| Synthesis methods | 13a | Describe the processes used to decide which studies were eligible for each synthesis (e.g. tabulating the study intervention characteristics and comparing against the planned groups for each synthesis (item #5)). | 7 |
|  | 13b | Describe any methods required to prepare the data for presentation or synthesis, such as handling of missing summary statistics, or data conversions. | 7 |
|  | 13c | Describe any methods used to tabulate or visually display results of individual studies and syntheses. | 7 |
|  | 13d | Describe any methods used to synthesize results and provide a rationale for the choice(s). If meta-analysis was performed, describe the model(s), method(s) to identify the presence and extent of statistical heterogeneity, and software package(s) used. | 7 |
|  | 13e | Describe any methods used to explore possible causes of heterogeneity among study results (e.g. subgroup analysis, meta-regression). | 7 |
|  | 13f | Describe any sensitivity analyses conducted to assess robustness of the synthesized results. | 7 |
| Reporting bias assessment | 14 | Describe any methods used to assess risk of bias due to missing results in a synthesis (arising from reporting biases). | 7 |
| Certainty assessment | 15 | Describe any methods used to assess certainty (or confidence) in the body of evidence for an outcome. |  |
| **RESULTS** | | |  |
| Study selection | 16a | Describe the results of the search and selection process, from the number of records identified in the search to the number of studies included in the review, ideally using a flow diagram. | 8 |
|  | 16b | Cite studies that might appear to meet the inclusion criteria, but which were excluded, and explain why they were excluded. | 8, Appendix 4 |
| Study characteristics | 17 | Cite each included study and present its characteristics. | 11-12 |
| Risk of bias in studies | 18 | Present assessments of risk of bias for each included study. | 13-15 |
| Results of individual studies | 19 | For all outcomes, present, for each study: (a) summary statistics for each group (where appropriate) and (b) an effect estimate and its precision (e.g. confidence/credible interval), ideally using structured tables or plots. | 15-16, 18-20 |
| Results of syntheses | 20a | For each synthesis, briefly summarise the characteristics and risk of bias among contributing studies. | n/a |
|  | 20b | Present results of all statistical syntheses conducted. If meta-analysis was done, present for each the summary estimate and its precision (e.g. confidence/credible interval) and measures of statistical heterogeneity. If comparing groups, describe the direction of the effect. | n/a |
|  | 20c | Present results of all investigations of possible causes of heterogeneity among study results. | n/a |
|  | 20d | Present results of all sensitivity analyses conducted to assess the robustness of the synthesized results. | n/a |
| Reporting biases | 21 | Present assessments of risk of bias due to missing results (arising from reporting biases) for each synthesis assessed. | n/a |
| Certainty of evidence | 22 | Present assessments of certainty (or confidence) in the body of evidence for each outcome assessed. |  |
| **DISCUSSION** | | |  |
| Discussion | 23a | Provide a general interpretation of the results in the context of other evidence. | 21 |
|  | 23b | Discuss any limitations of the evidence included in the review. | 21-22 |
|  | 23c | Discuss any limitations of the review processes used. | 22 |
|  | 23d | Discuss implications of the results for practice, policy, and future research. | 21-22 |
| **OTHER INFORMATION** | | |  |
| Registration and protocol | 24a | Provide registration information for the review, including register name and registration number, or state that the review was not registered. | 5 |
|  | 24b | Indicate where the review protocol can be accessed, or state that a protocol was not prepared. | 5 |
|  | 24c | Describe and explain any amendments to information provided at registration or in the protocol. |  |
| Support | 25 | Describe sources of financial or non-financial support for the review, and the role of the funders or sponsors in the review. | 23 |
| Competing interests | 26 | Declare any competing interests of review authors. | 23 |
| Availability of data, code and other materials | 27 | Report which of the following are publicly available and where they can be found: template data collection forms; data extracted from included studies; data used for all analyses; analytic code; any other materials used in the review. | 23 |

*From:*  Page MJ, McKenzie JE, Bossuyt PM, Boutron I, Hoffmann TC, Mulrow CD, et al. The PRISMA 2020 statement: an updated guideline for reporting systematic reviews. BMJ 2021;372:n71. doi: 10.1136/bmj.n71

For more information, visit: <http://www.prisma-statement.org/>

## Appendix 2 - Search strategies

**All searches run on 17/01/2025**

### PubMed

("Systematic Reviews as Topic"[Mesh] OR "Meta-Analysis as Topic"[Mesh] OR "Review Literature as Topic"[Mesh] OR "Systematic review"[tiab] OR "Systematic reviews"[tiab] OR "Systematic literature"[tiab] OR "Literature review"[tiab] OR "Literature reviews"[tiab] OR "Meta analysis"[tiab] OR Meta-analysis[tiab] OR Meta-analyses[tiab] OR "Meta analyses"[tiab] OR "Evidence synthesis"[tiab] OR "Evidence syntheses"[tiab] OR "Scoping review"[tiab] OR "Scoping reviews"[tiab] OR "Umbrella review"[tiab] OR "Umbrella reviews"[tiab] OR "Narrative review"[tiab] OR "Narrative reviews"[tiab] OR "Rapid review"[tiab] OR "Rapid reviews"[tiab])

AND

("Large language"[tiab] OR "Language models"[tiab] OR "Language model"[tiab] OR LLM[tiab] OR LLMs[tiab] OR Chatgpt[tiab] OR "Generative AI"[tiab] OR "Generative Artificial Intelligence"[tiab] OR GPT[tiab] OR GPT-3[tiab] OR GPT3[tiab] OR GPT-4[tiab] OR GPT4[tiab] OR LaMDA[tiab] OR LLaMA[tiab] OR LLaMa2[tiab] OR LLaMa2[tiab] OR BLOOM[tiab] OR Gemini[tiab] OR Claude[tiab] OR  "Claude 2"[tiab] OR Claude2[tiab] OR Bing-AI[tiab] OR "Vertex AI"[tiab] OR Perplexity.AI[tiab] OR Mistral[tiab])

AND

(“randomized controlled trial”[pt] OR “controlled clinical trial”[pt] OR randomized[tiab] OR randomised[tiab] OR placebo[tiab] OR randomly[tiab] OR trial[tiab] OR groups[tiab] OR Crossover[tiab] OR "Comparative Study"[pt] OR "Evaluation Study"[pt] OR "Epidemiologic Studies"[Mesh] OR “case-control studies”[Mesh] OR “Cohort Studies”[Mesh] OR “case control”[tiab] OR Cohort[tiab] OR “Follow up”[tiab] OR Observational[tiab] OR Longitudinal[tiab] OR Prospective[tiab] OR Retrospective[tiab] OR “cross sectional”[tiab] OR “Cross-Sectional Studies”[Mesh] OR Investigated[tiab] OR Evaluated[tiab] OR Impact[tiab] OR Analysis[tiab] OR Statistics[tiab] OR Data[tiab] OR "statistics and numerical data"[sh] OR "epidemiology"[sh] OR Test[tiab] OR Tested[tiab] OR Evaluate[tiab] OR Evaluated[tiab] OR Sensitivity[tiab] OR Specificity[tiab])

### Embase (via Elsevier)

('systematic review (topic)'/exp/mj OR 'meta analysis (topic)'/exp/mj OR 'Systematic review':ti,ab OR 'Systematic reviews':ti,ab OR 'Systematic literature':ti,ab OR 'Literature review':ti,ab OR 'Literature reviews':ti,ab OR 'Meta analysis':ti,ab OR Meta-analysis:ti,ab OR Meta-analyses:ti,ab OR 'Meta analyses':ti,ab OR 'Evidence synthesis':ti,ab OR 'Evidence syntheses':ti,ab OR 'Scoping review':ti,ab OR 'Scoping reviews':ti,ab OR 'Umbrella review':ti,ab OR 'Umbrella reviews':ti,ab OR 'Narrative review':ti,ab OR 'Narrative reviews':ti,ab OR 'Rapid review':ti,ab OR 'Rapid reviews':ti,ab)

AND

('Large language':ti,ab OR 'Language models':ti,ab OR 'Language model':ti,ab OR LLM:ti,ab OR LLMs:ti,ab OR Chatgpt:ti,ab OR 'Generative AI':ti,ab OR 'Generative Artificial Intelligence':ti,ab OR GPT:ti,ab OR GPT-3:ti,ab OR GPT3:ti,ab OR GPT-4:ti,ab OR GPT4:ti,ab OR LaMDA:ti,ab OR LLaMA:ti,ab OR LLaMa2:ti,ab OR LLaMa2:ti,ab OR BLOOM:ti,ab OR Gemini:ti,ab OR Claude:ti,ab OR 'Claude 2':ti,ab OR Claude2:ti,ab OR Bing-AI:ti,ab OR 'Vertex AI':ti,ab OR Perplexity:ti,ab OR Mistral:ti,ab)

AND

(random* OR factorial OR crossover OR placebo OR blind OR blinded OR assign OR assigned OR allocate OR allocated OR 'crossover procedure'/exp OR 'double-blind procedure'/exp OR 'randomized controlled trial'/exp OR 'single-blind procedure'/exp OR 'epidemiology'/exp OR 'controlled study'/exp OR 'cohort analysis'/exp OR "case control":ti,ab OR Cohort:ti,ab OR "Follow up":ti,ab OR Observational:ti,ab OR longitudinal:ti,ab OR Prospective:ti,ab OR retrospective:ti,ab OR "cross sectional":ti,ab OR 'Cross-Sectional Studies'/exp OR Investigated:ti,ab OR Analysis:ti,ab OR Statistics:ti,ab OR Data:ti,ab OR Test:ti,ab OR Tested:ti,ab OR Evaluate:ti,ab OR Evaluated:ti,ab OR Sensitivity:ti,ab OR Specificity:ti,ab)

### Scopus

("Systematic Reviews as Topic" OR "Meta-Analysis as Topic" OR "Review Literature as Topic" OR "Systematic review" OR "Systematic reviews" OR "Systematic literature" OR "Literature review" OR "Literature reviews" OR "Meta analysis" OR Meta-analysis OR Meta-analyses OR "Meta analyses" OR "Evidence synthesis" OR "Evidence syntheses" OR "Scoping review" OR "Scoping reviews" OR "Umbrella review" OR "Umbrella reviews" OR "Narrative review" OR "Narrative reviews" OR "Rapid review" OR "Rapid reviews")

AND

("Large language" OR "Language models" OR "Language model" OR LLM OR LLMs OR Chatgpt OR "Generative AI" OR "Generative Artificial Intelligence" OR GPT OR GPT-3 OR GPT3 OR GPT-4 OR GPT4 OR LaMDA OR LLaMA OR LLaMa2 OR LLaMa2 OR BLOOM OR Gemini OR Claude OR "Claude 2" OR Claude2 OR Bing-AI OR "Vertex AI" OR Perplexity OR Mistral)

AND

(Trial OR randomized OR randomised OR placebo OR randomly OR groups OR "Epidemiologic Studies" OR Epidemiological OR "case-control studies" OR "Cohort Studies" OR "case control" OR Cohort OR "Follow up" OR Observational OR longitudinal OR Prospective OR retrospective OR "cross sectional" OR "Cross-Sectional Studies" OR Investigated OR Analysis OR Statistics OR Data OR Test OR Tested OR Evaluate OR Evaluated OR Sensitivity OR Specificity)

### Web of Science core collection

("Systematic Reviews as Topic" OR "Meta-Analysis as Topic" OR "Review Literature as Topic" OR "Systematic review" OR "Systematic reviews" OR "Systematic literature" OR "Literature review" OR "Literature reviews" OR "Meta analysis" OR Meta-analysis OR Meta-analyses OR "Meta analyses" OR "Evidence synthesis" OR "Evidence syntheses" OR "Scoping review" OR "Scoping reviews" OR "Umbrella review" OR "Umbrella reviews" OR "Narrative review" OR "Narrative reviews" OR "Rapid review" OR "Rapid reviews")

AND

("Large language" OR "Language models" OR "Language model" OR LLM OR LLMs OR Chatgpt OR "Generative AI" OR "Generative Artificial Intelligence" OR GPT OR GPT-3 OR GPT3 OR GPT-4 OR GPT4 OR LaMDA OR LLaMA OR LLaMa2 OR LLaMa2 OR BLOOM OR Gemini OR Claude OR "Claude 2" OR Claude2 OR Bing-AI OR "Vertex AI" OR Perplexity OR Mistral)

AND

(Trial OR randomized OR randomised OR placebo OR randomly OR groups OR "Epidemiologic Studies" OR Epidemiological OR "case-control studies" OR "Cohort Studies" OR "case control" OR Cohort OR "Follow up" OR Observational OR longitudinal OR Prospective OR retrospective OR "cross sectional" OR "Cross-Sectional Studies" OR Investigated OR Analysis OR Statistics OR Data OR Test OR Tested OR Evaluate OR Evaluated OR Sensitivity OR Specificity)

### Business Source Ultimate (via Ebsco)

("Systematic Reviews as Topic" OR "Meta-Analysis as Topic" OR "Review Literature as Topic" OR "Systematic review" OR "Systematic reviews" OR "Systematic literature" OR "Literature review" OR "Literature reviews" OR "Meta analysis" OR Meta-analysis OR Meta-analyses OR "Meta analyses" OR "Evidence synthesis" OR "Evidence syntheses" OR "Scoping review" OR "Scoping reviews" OR "Umbrella review" OR "Umbrella reviews" OR "Narrative review" OR "Narrative reviews" OR "Rapid review" OR "Rapid reviews")

AND

("Large language" OR "Language models" OR "Language model" OR LLM OR LLMs OR Chatgpt OR "Generative AI" OR "Generative Artificial Intelligence" OR GPT OR GPT-3 OR GPT3 OR GPT-4 OR GPT4 OR LaMDA OR LLaMA OR LLaMa2 OR LLaMa2 OR BLOOM OR Gemini OR Claude OR "Claude 2" OR Claude2 OR Bing-AI OR "Vertex AI" OR Perplexity OR Mistral)

AND

(Trial OR randomized OR randomised OR placebo OR randomly OR groups OR "Epidemiologic Studies" OR Epidemiological OR "case-control studies" OR "Cohort Studies" OR "case control" OR Cohort OR "Follow up" OR Observational OR longitudinal OR Prospective OR retrospective OR "cross sectional" OR "Cross-Sectional Studies" OR Investigated OR Analysis OR Statistics OR Data OR Test OR Tested OR Evaluate OR Evaluated OR Sensitivity OR Specificity)

## Appendix 2: Risk of bias

#### Table S1: Comparison between original and modified version of QUADAS-2

|  | QUADAS-2 original | QUADAS-2 modified |
| --- | --- | --- |
| Domain | Domain 1: patient selection | Domain 1: Review selection |
| Description | Describe methods of patient selection: Describe included patients (prior testing, presentation, intended use of index test and setting): | Describe how the reviews used in the study were selected and any reviews that were excluded. |
| Signalling question | Was a consecutive or random sample of patients enrolled? | Was a consecutive or random sample of review/s selected? |
| Signalling question | Did the study avoid inappropriate exclusions? | Did the study avoid inappropriate exclusions of reviews? |
| 1A: Risk of bias | Could the selection of patients have introduced bias? | Could the selection of reviews have introduced bias? |
| 1B: Concerns regarding applicability | Are there concerns that the included patients do not match the review question? | Is there concern that the study findings in the evaluations are not applicable to any type of review? (e.g. only searching for a single study type, or sample size too small) |
| Domain | Domain 2: Index test | Domain 2: GenAI |
| Description | Describe the index test and how it was conducted and interpreted: | Describe how the Gen AI tool was used and evaluated. |
| Signalling question | Were the index test results interpreted without knowledge of the results of the reference standard? | Were the Gen AI/LLM tasks run without any knowledge of the results from the original review/s? |
| Signalling question | If a threshold was used, was it pre-specified? | If prompts were used, were they pre-specified? |
| 2A: Risk of bias | Could the conduct or interpretation of the index test have introduced bias? | Could the conduct or interpretation of the GenAI test have introduced bias? |
| 2B: Concerns regarding applicability | Are there concerns that the index test, its conduct, or interpretation differ from the review question? | Is there concern that the GenAI/LLM tool is not capable of being use by a standard review team? |
| Domain | Domain 3: Reference standard | Domain 3: Human task |
| Description | Describe the reference standard and how it was conducted and interpreted. | Describe how the tasks done by the humans were conducted and evaluated. |
| Signalling question | Is the reference standard likely to correctly classify the target condition? | Were the tasks done in the original review to an adequate standard? |
| Signalling question | Were the reference standard results interpreted without knowledge of the results of the index test? | Were the tasks done in the original review/s done without knowledge of the results of the GenAI tasks? |
| 3A: Risk of bias: | Could the reference standard, its conduct, or its interpretation have introduced bias? | Could the tasks from the original review/s, its conduct, or its interpretation have introduced bias? |
| 3B: Concerns regarding applicability | Are there concerns that the target condition as defined by the reference standard does not match the review question? | Is there concern that the review task/s from the original review are not capable of being replicated by a standard SR team? |
| Domain | Domain 4: Flow and timing | Domain 4: Differences |
| Description | Describe any patients who did not receive the index test(s) and/or reference standard or who were excluded from the 2x2 table (refer to flow diagram): Describe the time interval and any interventions between index test(s) and reference standard. | Describe any differences between the review tasks done by the GenAI tools and the humans (e.g., topic, searcher skills, or time frames) |
| Signalling question | Was there an appropriate interval between index test(s) and reference standard? | Were the GenAI task/s done on the same topics as the original review/s? |
| Signalling question | Did all patients receive a reference standard? | Were all task/s attempted by the GenAI tool on all the reviews? |
| Signalling question | Did all patients receive the same reference standard? | Were review task/s from the original review/s done by the same team, or teams with equivalent skill levels? |
| Signalling question | Were all patients included in the analysis? | Were all review/s and/or task/s studies included in the analysis? |
| 4: Risk of bias | Could the patient flow have introduced bias? | Could any differences between the GenAI and human conduct have introduced bias? |

## Appendix 3: List of excluded studies

1. LWDA 2023 - Lernen, Wissen, Daten, Analysen, Conference Proceedings. **Exclude - wrong comparator**

**(no humans)**

2. Adam GP, et al. Literature search sandbox: a large language model that generates search queries for systematic reviews. **Exclude - wrong comparator**

**Tested against a cleaned version of the search, not the search itself. Also only searched PubMed, not the full range of databases from the original reviews.**

3. Akinseloyin O, et al. A Novel Question-Answering Framework for Automated Abstract Screening Using Large Language Models. **Exclude - wrong publication type**

**(preprint)**

4. Akinseloyin O, et al. A question-answering framework for automated abstract screening using large language models. **Exclude - wrong outcomes**

**Did not report on inclusion or exclusions from screening.**

5. Al-Mahayri Z. Generative AI as a catalyst for precision medicine research: Bridging potential with reality. **Exclude - wrong publication type**

6. Alchokr R, et al. Supporting Systematic Literature Reviews Using Deep-Learning-Based Language Models. **Exclude - wrong intervention**

**BERT is based on transformer models, so technically not generative.**

7. Alshami A, et al. Harnessing the Power of ChatGPT for Automating Systematic Review Process: Methodology, Case Study, Limitations, and Future Directions. **Exclude - wrong comparison**

**(humans used GenAI to do the review, they did not do it with and then without GenAI)**

8. Ambalavanan AK, et al. Using the contextual language model BERT for multi-criteria classification of scientific articles. **Exclude - wrong population**

9. Aum S, et al. srBERT: automatic article classification model for systematic review using BERT. **Exclude - wrong comparator**

**(no humans)**

10. Baisley W, et al. MSR49 Non-Systematic Literature Reviews: Can AI Enhance Current Methods?: **Exclude - wrong publication type**

11. Barsby J, et al. Pilot study on large language models for risk-of-bias assessments in systematic reviews: A(I) new type of bias?: **Exclude - wrong publication type**

**(letter, not peer reviewed)**

12. Berger-Tal O, et al. Leveraging AI to improve evidence synthesis in conservation.: **Exclude - wrong study type**

**(not a comparative study)**

13. Bond A, et al. Using an Artificial intelligence chatbot to critically review the scientific literature on the use of Artificial intelligence in Environmental Impact Assessment. **Exclude - wrong comparator**

**(no humans)**

14. Budau L, et al. Fully Automated Scholarly Search for Biomedical Systematic Literature Reviews. **Exclude - wrong comparator**

**Seemed to only collect 1000 search results, not all search results.**

15. Cai X, et al. Utilizing ChatGPT to select literature for meta-analysis shows workload reduction while maintaining a similar recall level as manual curation. **Exclude - wrong publication type**

16. Cao C, et al. Prompting is all you need: LLMs for systematic review screening. **Exclude - wrong publication type**

**(preprint)**

17. Cheloff AZ, et al. Publicly Available Generative Artificial Intelligence Programs Are Currently Unsuitable for Performing Meta-Analyses. **Exclude - wrong publication type**

18. Cheloff AZ, et al. POTENTIAL OF GENERATIVE AI IN META-ANALYSIS: AUTOMATING LITERATURE REVIEW AND DATA EXTRACTION. **Exclude - wrong comparator**

19. Cheng R, et al. Can Machine Learning Assist With Large Scale Medical Literature Review?: **Exclude - wrong publication type**

**Conference abstract.**

20. Damentko M, et al. MSR66 Natural Language Processing to Support the Abstract Selection During Systematic Literature Review. **Exclude - wrong publication type**

21. Delgado-Chaves FM, et al. Transforming literature screening: The emerging role of large language models in systematic reviews. **Exclude: wrong comparator**

**Compared different LLMs to each other, to see which performed best using retrieval-augmented generation (RAG) which requires you to identiy the relevant papers first, then feed these into the LLM. "We utilized lists of identified articles from three existing SRs, feeding them separately to the LLMs."**

22. Dennstädt F, et al. Title and abstract screening for literature reviews using large language models: an exploratory study in the biomedical domain. **Exclude - wrong study type**

**Was experimental, and changed parameters to determine effect, did not seem to have a fixed test.**

23. Du J, et al. Machine learning models for abstract screening task - A systematic literature review application for health economics and outcome research. **Exclude - wrong intervention**

**(not a standard SR task as "training" people on 89-90% of the articles to be screened is not standard)**

24. Đukić M, et al. Towards the Utilization of AI-Powered Assistance for Systematic Literature Review. **Exclude - wrong comparator**

**Compares tools to each other, not to humans.**

25. Esposito C, et al. [Human vs. ChatGPT. Is it possible obtain comparable results in the analysis of a scientific systematic review?]. **Exclude - wrong study type**

**Not a comparative study, was a development study where they performed iterations of the prompt until a good result was obtained.**

26. Feng Y, et al. Automated medical literature screening using artificial intelligence: a systematic review and meta-analysis.: **Exclude - wrong study type**

**(systematic review)**

27. Fernández-López J, et al. Using Artificial Intelligence-Based Tools to Improve the Literature Review Process: Pilot Test with the Topic “Hybrid Meat Products”. **Exclude - wrong outcomes**

**Did not report on recall, precision or time.**

28. García-Torres D, et al. Enhancing Clinical Reasoning with Virtual Patients: A Hybrid Systematic Review Combining Human Reviewers and ChatGPT. **Exclude - wrong outcomes**

**Looked at whether GenAI identified key elemtns in a study, not whether it correct;ly included or excluded it.**

29. Gartlehner G, et al. Data Extraction for Evidence Synthesis Using a Large Language Model: A Proof-of-Concept Study. **Exclude - repeated data**

30. Giobergia F, et al. Large Language Models-aided Literature Reviews: A Study on Few-Shot Relevance Classification. **Exclude - wrong comparator**

**Compared LLM models with each other to determine which may be best for screening.**

31. Guerra I, et al. MSR92 Can Artificial Intelligence (AI) Large Language Models (LLMS) Such as Generative Pre-Trained Transformer (GPT) Be Used to Automate Literature Reviews?: **Exclude - wrong publication type**

32. Guo F, et al. SciMine: An Efficient Systematic Prioritization Model Based on Richer Semantic Information. **Exclude - wrong comparator**

**(no humans)**

33. Haman M, et al. Using ChatGPT to conduct a literature review.: **Exclude - wrong study type**

**(not a comparative study)**

34. Hanegraaf P, et al. MSR5 Accuracy of Automated Data Extraction for Systematic Literature Reviews. **Exclude - wrong study type**

35. Hasny M, et al. BERT for Complex Systematic Review Screening to Support the Future of Medical Research. **Exclude - wrong study type**

36. Hellstrom W, et al. CAN ARTIFICIAL INTELLIGENCE GENERATE A SUPERIOR INTRODUCTION TO A SYSTEMATIC REVIEW?: **Exclude - wrong population**

37. Herbst P, et al. Accelerating literature screening for systematic literature reviews with Large Language Models – development, application, and first evaluation of a solution. **Exclude - wrong comparator**

**(doesn't compare to humans)**

38. Hill JE, et al. Methods for using Bing's AI-powered search engine for data extraction for a systematic review. **Exclude - wrong study type**

**Not a comparative study.**

39. Hudgens S, et al. Screening Articles in a Qualitative Literature Review Using Large Language Models: A Comparison of GPT Versus Open Source, Trained Models Against Expert Researcher Screening. **Exclude - wrong publication type**

**Conference abstract.**

40. Huotala A, et al. The Promise and Challenges of Using LLMs to Accelerate the Screening Process of Systematic Reviews. **Exclude - wrong population**

**Did not use a real review task, they instead compiled a set of articles to screen that resembled one.**

41. Kartchner D, et al. Zero-Shot Information Extraction for Clinical Meta-Analysis using Large Language Models. **Exclude - wrong study type**

42. Kataoka Y, et al. Development of meta-prompts for Large Language Models to screen titles and abstracts for diagnostic test accuracy reviews. **Exclude - wrong study type**

**(preprint)**

43. Kebede M, et al. In-depth evaluation of machine learning methods for semi-automating article screening in a systematic review of mechanistic literature.: **Exclude - wrong intervention**

**(not Gen AI/LLM)**

44. Khan MA, et al. Collaborative Large Language Models for Automated Data Extraction in Living Systematic Reviews. **Exclude - wrong publication type**

**Preprint.**

45. Khlaif ZN, et al. The Potential and Concerns of Using AI in Scientific Research: ChatGPT Performance Evaluation. **Exclude - wrong population**

**(was used, in part, to wirte a literature review section in a manuscript, was not used for a standard evidence synthesis)**

46. Knafou J, et al. Ensemble of deep learning language models to support the creation of living systematic reviews for the COVID-19 literature. **Exclude - wrong publication type**

**(preprint)**

47. Kohandel Gargari O, et al. Enhancing title and abstract screening for systematic reviews with GPT-3.5 turbo.: **Exclude - wrong publication type**

**(letter, not peer reviewed)**

48. Kuitunen I, et al. Evaluating the Performance of ChatGPT-4o in Risk of Bias Assessments. **Exclude - wrong publication type**

**This is a letter to the editor.**

49. Landschaft A, et al. Implementation and evaluation of an additional GPT-4-based reviewer in PRISMA-based medical systematic literature reviews. **Exclude - wrong population**

**Did not test on a complete review task, only tested on the top 1000 extracted from Google Scholar.**

50. Langham J, et al. MSR80 AI-Enabled Risk of Bias Assessment of RCTs in Systematic Reviews: A Case Study. **Exclude - wrong publication type**

**(conference abstract so not peer reviewed)**

51. Lee K, et al. AID-SLR: A Generative Artificial Intelligence-Driven Automated System for Systematic Literature Review. **Exclude - wrong publication type**

**Preprint.**

52. Lessani MN, et al. Leveraging large language models for systematic reviewing: A case study using HIV medication adherence research. **Exclude - wrong publication type**

**Preprint.**

53. Li M, et al. Evaluating the Effectiveness of Large Language Models in Abstract Screening: A Comparative Analysis. **Exclude - wrong publication type**

**(preprint)**

54. Li M, et al. Evaluating the effectiveness of large language models in abstract screening: a comparative analysis. **Exclude - wrong study type**

**Not a comparative study, was am experimental/development study where they tested a number of approaches/GenAI models and reported the results from each.**

55. Li T, et al. The Heap, the Hype, the Reality: Generative Pretrained Transformer for Systematic Reviews.: **Exclude - wrong study type**

**(not a comparative study, editorial)**

56. Liu R, et al. Improving Efficiency of Living Systematic Literature Reviews (SLR) with Artificial Intelligence (AI): Assisted Extraction of Population, Intervention/Comparator, Outcome, and Study Design (P-I/C-O-S). **Exclude - wrong study type**

**(conference abstract, also not enough data)**

57. Livberber T. Toward non-human-centered design: designing an academic article with ChatGPT. **Exclude - wrong population**

**(not used in any SR tasks, used to write a research article)**

58. Luedtke NF, et al. Automated extraction of randomized controlled trial data using large language models: a pilot study with vedolizumab meta-analysis. **Exclude - wrong study type**

**(conference abstract, not enough data)**

59. Luo R, et al. Evaluating the Efficacy of Large Language Models for Systematic Review and Meta-Analysis Screening. **Exclude - wrong publication type**

**Preprint.**

60. Mackenzie E, et al. GPT3 Meets PubMed: A Novel Approach to Meta-Analysis Using a Large Language Model to Crowdsource Migraine Medication Reviews. **Exclude - wrong publication type**

**(conference abstract so not peer reviewed)**

61. Mahuli SA, et al. Application ChatGPT in conducting systematic reviews and meta-analyses. **Exclude - wrong study type**

**(not a comparative study, they more just tried ChatGPT out for a few SR tasks and gave their opinion on how it performed)**

62. Manning C, et al. Streamlining Science: Recreating Systematic Literature Reviews with AI-Powered Decision Tools. **Exclude - wrong publication type**

**(conference abstract, so not peer reviewed)**

63. Masinde M. Enhancing Systematic Literature Reviews using LDA and ChatGPT: Case of Framework for Smart City Planning. **Exclude - wrong outcomes**

**Did not report accuracy data.**

64. Masoumi S, et al. Natural language processing (NLP) to facilitate abstract review in medical research: the application of BioBERT to exploring the 20-year use of NLP in medical research. **Exclude - inadequate data**

65. Moreno-Garcia C, et al. A novel application of machine learning and zero-shot classification methods for automated abstract screening in systematic reviews. **Exclude - wrong intervention**

**(not Gen AI/LLM)**

66. Mostafapour M, et al. Evaluating Literature Reviews Conducted by Humans Versus ChatGPT: Comparative Study. **Exclude - wrong outcomes**

**Seemed to be a qualitative comparison of review quality, not actual numbers of errors made.**

67. Mutinda FW, et al. Automatic data extraction to support meta-analysis statistical analysis: a case study on breast cancer. **Exclude - wrong comparator**

**(not an SR task)**

68. Na CB, et al. Pilot study on the accuracy of ChatGPT in article screening for systematic reviews in gastroenterology. **Exclude - wrong publication type**

**Conference abstract.**

69. Nashwan AJ, et al. Streamlining Systematic Reviews: Harnessing Large Language Models for Quality Assessment and Risk-of-Bias Evaluation. **Exclude - wrong study type**

**(editorial)**

70. Norman C, et al. A distantly supervised dataset for automated data extraction from diagnostic studies. **Exclude - wrong population**

**(did not perform an SR task)**

71. Ohra S, et al. Quality Appraisal of Randomized Controlled Trials Using Robins 2.0 Tool: A Case Study on Comparing the Performance of ChatGPTv4.0 With a Human Reviewer. **Exclude - wrong publication type**

**Conference abstract.**

72. Ötles E, et al. Evaluating the efficacy of a large language model in screening ophthalmology articles for systematic reviews. **Exclude - wrong publication type**

**Conference abstract.**

73. Panayi A, et al. Machine learning to automate data extraction for systematic literature reviews. **Exclude - wrong population**

**(did not perform an SR task)**

74. Panayi A, et al. Evaluation of a prototype machine learning tool to semi-automate data extraction for systematic literature reviews. **Exclude - wrong population**

**(did not perform an SR task)**

75. Pitre T, et al. ChatGPT for assessing risk of bias of randomized trials using the RoB 2.0 tool: A methods study. **Exclude - wrong publication type**

**(preprint)**

76. Platt M, et al. Effectiveness of Generative Artificial Intelligence for Scientific Content Analysis. **Exclude - wrong population**

**Only conducted part of the data extraction task, only extracted country, study type and participant numbers.**

77. Qin X, et al. Natural language processing was effective in assisting rapid title and abstract screening when updating systematic reviews. **Exclude - inadequate data**

**(did not report results in enough detail)**

78. Raja H, et al. Automated Category and Trend Analysis of Scientific Articles on Ophthalmology Using Large Language Models: Development and Usability Study. **Exclude - wrong population**

79. Rathi H, et al. A Comparative Analysis of Large Language Models (LLM) Utilised in Systematic Literature Review. **Exclude - wrong study type**

80. Rathi H, et al. USE OF LARGE LANGUAGE MODEL (LLM) FOR FULL-TEXT SCREENING IN SYSTEMATIC LITERATURE REVIEWS: A COMPARATIVE ANALYSIS. **Exclude - wrong publication type**

**Conference abstract.**

81. Reason T, et al. MSR46 Breaking Through Limitations: Enhanced Systematic Literature Reviews With Large Language Models. **Exclude - wrong study type**

82. Reason T, et al. Disrupting Health Economics: Automating Network Meta-Analyses With AI and Large Language Models. **Exclude - wrong study type**

83. Reed RB, et al. Assessing the Quality of Biomedical Boolean Search Strings Generated by Prompted and Unprompted Models Using ChatGPT: A Pilot Study. **Exclude - wrong outcomes**

**Did not report recall or precision.**

84. Roberts RH, et al. Comparative study of ChatGPT and human evaluators on the assessment of medical literature according to recognised reporting standards. **Exclude - wrong population**

85. Royer J, et al. MSR131 Prospects for Automation of Systemic Literature Reviews (SLRs) With Artificial Intelligence and Natural Language Processing. **Exclude - wrong publication type**

**(conference abstract)**

86. Ruksakulpiwat S, et al. Using ChatGPT in Medical Research: Current Status and Future Directions. **Exclude - wrong study type**

87. Ruksakulpiwat S, et al. Assessing the Efficacy of ChatGPT Versus Human Researchers in Identifying Relevant Studies on mHealth Interventions for Improving Medication Adherence in Patients With Ischemic Stroke When Conducting Systematic Reviews: Comparative Analysis. **Exclude - inadequate data**

**(did not provide a reference standard measure to compare against, therefore can not determine how much of the evidence (relevant studies) was found.)**

88. Shah-Mohammadi F, et al. Large Language Model-Based Architecture for Automatic Outcome Data Extraction to Support Meta-Analysis. **Exclude - wrong study type**

89. Souifi L, et al. Towards the Use of AI-Based Tools for Systematic Literature Review. **Exclude - wrong comparator**

**(not compared to humans doing an SR task)**

90. Spillias S, et al. Human-AI collaboration to identify literature for evidence synthesis. **Exclude - wrong study type**

**Not a comparative study, was an experimental/development study where screening criteria fed to the AI was iteratively tested until inter-rater reliability scores were similar to those found between the individual human raters.**

91. Sun Z, et al. How good are large language models for automated data extraction from randomized trials?: **Exclude - wrong publication type**

**(preprint)**

92. Šuster S, et al. Zero- and few-shot prompting of generative large language models provides weak assessment of risk of bias in clinical trials. **Exclude - wrong study type**

**Was an experimental/development study where they tested a number of GenAI models for assessing RoB2 and reported on the results of each.**

93. Syriani, et al. Assessing the Ability of ChatGPT to Screen Articles for Systematic Reviews. **Exclude - wrong publication type**

**(preprint)**

94. Syriani E, et al. Screening articles for systematic reviews with ChatGPT. **Exclude - wrong study type**

**Not really a comparative study, it is an experimental where they did lots of things until they found things that worked.**

95. Tang Y, et al. Large Language Model in Medical Information Extraction from Titles and Abstracts with Prompt Engineering Strategies: A Comparative Study of GPT-3.5 and GPT-4. **Exclude - wrong publication type**

96. Teperikidis E, et al. Does the long-term administration of proton pump inhibitors increase the risk of adverse cardiovascular outcomes? A ChatGPT powered umbrella review. **Exclude - wrong study type**

97. Teperikidis L, et al. Validating ChatGPT's role in systematic reviews and meta-analyses: A case study on GLP-1 receptor agonists and all-cause mortality. **Exclude - wrong publication type**

**Letter to the editor.**

98. Thorlund K, et al. Screening oncology articles in a qualitative literature review using large language models: A comparison of GPT4 versus fine-tuned open source models using expertannotated data. **Exclude - wrong publication type**

**Conference abstract.**

99. Tran VT, et al. Sensitivity, specificity and avoidable workload of using a large language models for title and abstract screening in systematic reviews and meta-analyses. **Exclude - wrong publication type**

100. Treviño-Juarez A. Assessing Risk of Bias Using ChatGPT-4 and Cochrane ROB2 Tool. **Exclude - wrong study type**

**(not a comparative study)**

101. van Dijk S, et al. Artificial intelligence in systematic reviews: promising when appropriately used.: **Exclude - wrong intervention**

**(not Gen AI/LLM)**

102. Wang Q, et al. PICO entity extraction for preclinical animal literature. **Exclude - wrong comparator**

103. Wang Q, et al. Risk of bias assessment in preclinical literature using natural language processing. **Exclude - wrong intervention**

**(not testing whether it gets the RoB assessment correct, they are testing to see if it has RoB information)**

104. Wang S, et al. Neural Rankers for Effective Screening Prioritisation in Medical Systematic Review Literature Search. **Exclude - wrong study type**

105. Wang S, et al. Automated MeSH term suggestion for effective query formulation in systematic reviews literature search. **Exclude - wrong comparator**

**(not compared to humans doing an SR task)**

106. Wang S, et al. Zero-Shot Generative Large Language Models for Systematic Review Screening Automation. **Exclude - wrong comparator**

**(did not compare to humans)**

107. Wang Z, et al. Using GPT-4 to write a scientific review article: a pilot evaluation study. **Exclude - wrong publication type**

**(preprint)**

108. Whitton J, et al. Automated tabulation of clinical trial results: A joint entity and relation extraction approach with transformer-based language representations. **Exclude - wrong comparator**

**(not compared to humans doing an SR task)**

109. Wilson E, et al. Screening for in vitro systematic reviews: a comparison of screening methods and training of a machine learning classifier.: **Exclude - wrong intervention**

**(not Gen AI/LLM)**

110. Yang F, et al. Identifying key elements for evidence-base medicine using pretrained model and graph convolution network. **Exclude - wrong comparator**

**(not compared to a sample from evidence synthesis/systematic reviews)**

111. Yu F, et al. An Empirical Study Evaluating ChatGPT's Performance in Generating Search Strategies for Systematic Reviews. **Exclude - wrong outcomes**

**Used PRESS to determine if searches were good, did not report recall or preision.**

112. Zimmerman. Leveraging Large Language Models

for Literature Review Tasks - A Case Study

Using ChatGPT. In 3rd International Conference on Advanced Research in Technologies, Information, Innovation and Sustainability, ARTIIS 2023. **Exclude - wrong population**

**(does not conduct a proper SR task)**
